# Development of competitive lateral flow immunoassay coupled with silver enhancement for simple and sensitive salivary cortisol detection

**DOI:** 10.17179/excli2018-1824

**Published:** 2018-12-21

**Authors:** Amara Apilux, Sirirat Rengpipat, Wilasinee Suwanjang, Orawon Chailapakul

**Affiliations:** 1 Department of Clinical Chemistry, Faculty of Medical Technology, Mahidol University, 999 Phutthamonthon 4 Road, Salaya, Nakhon Pathom 73170, Thailand; 2 Department of Microbiology, Faculty of Science, Chulalongkorn University, 254 Phayathai Road, Patumwan, Bangkok 10330, Thailand; 3 Center for Research and Innovation, Faculty of Medical Technology, Mahidol University, 999 Phutthamonthon 4 Road, Salaya, Nakhon Pathom 73170, Thailand; 4 Electrochemistry and Optical Spectroscopy Center of Excellence (EOSCE), Department of Chemistry, Faculty of Science, Chulalongkorn University, 254 Phayathai Road, Patumwan, Bangkok 10330, Thailand; 5 Department of Chemistry, Faculty of Science, Chulalongkorn University, 254 Phayathai Road, Patumwan, Bangkok 10330, Thailand

**Keywords:** cortisol, lateral flow immunoassay, silver enhancement, gold nanoparticles

## Abstract

Cortisol is known as a stress biomarker. The measurement of cortisol levels is an early warning indicator for health conditions and diagnosis of stress-related diseases. Herein, a lateral flow immunoassay using a gold nanoparticle label with a silver enhancement system was developed for the simple, sensitive and rapid detection of cortisol. The developed assay was based on a competitive platform of which cortisol-BSA conjugate was immobilized at the test zone to compete with an analyte. The quantitative analysis was performed using gold nanoparticles (AuNPs) as signal labeling. Sequentially, the silver enhancement solution was applied in order to enhance the sensitivity of the assay with the results easily seen by the naked eye. Using this system, the limit of detection (LOD) was found to be 0.5 ng/mL with a 3.6 fold more sensitive detection than without the enhancement system (LOD = 1.8 ng/mL). The salivary cortisol analysis was in the range of 0.5-150 ng/mL (R^2^ = 0.9984), which is in the clinical acceptable range. For the semi-quantitative analysis, the intensity color of the results was analyzed using an image processing program. The proposed method was successfully applied to detect cortisol in saliva. In addition, the results from our method also complied with the ones of those obtained by using the commercial enzyme-linked immunosorbent assay (ELISA). This developed assay offers great promise for a non-invasive screening test of salivary cortisol.

## Introduction

In an era of globalization, several factors are likely to influence increased stress such as a growing economy, competition in society, and medical disorders. The subject of stress has become a serious concern because stress is a factor regarding many health problems. Cortisol (Kaushik et al., 2014[15], Hellhammer et al., 2009[13]), a steroid hormone, is the end product of the hypothalamic pituitary adrenal (HPA) axis. Cortisol secretion has a circadian rhythm with the highest levels in the morning period and the lowest levels at night. Stress causes an abnormal increase in the level of cortisol, which suppresses the immune system and inflammatory pathways. Moreover, long-term chronic stress relates to the exposure of constant cortisol adaptation. The symptoms include impaired cognition, accumulation of abdominal fat, and decreased thyroid function. Therefore, cortisol is considered a promising biomarker for stress. In addition, the excess cortisol levels contribute to the development of Cushing's diseases (Belaya et al., 2012[6]; Gatti et al., 2009[11]) while the decreased cortisol levels lead to Addison's diseases (Gatti et al., 2009[11]). Therefore, cortisol measurement has gained widespread attention for health monitoring, diagnostics treatment, and follow-ups of the stress-linked diseases as well as cortisol-related diseases. 

Blood cortisol is most commonly utilized in the clinical laboratory (Gatti et al., 2009[11]). However, cortisol is bound to transport the proteins globulin or transcortin, and albumin. The free cortisol which is the active part in a serum was only about 1-2. Alternatively, salivary cortisol has been shown to closely correlate well with the free cortisol in a serum (Lippi et al., 2009[17]). Moreover, the levels of them are independent of salivary enzymes (Hellhammer et al., 2009[13]). Therefore, the measurement of salivary cortisol provides an opportunity to detect cortisol for stress responses. 

In recent years, several techniques have been applied for measurement of cortisol. Gas chromatography/mass spectroscopy (Wood et al., 2008[29]) and liquid chromatography/mass spectroscopy (De Palo et al., 2009[10]) have been widely used in a clinical laboratory. Although, these methods provide high sensitivity and accuracy, they are time-consuming as it includes sample preparation procedure, requires skilled operators, laboratory set up, and has a high operating cost. Currently, immunoassays based on various detection methods including; surface plasmon resonance (Stevens et al., 2008[22]; Mitchell et al., 2009[18]), electrochemical (Arya et al., 2010[3], 2011[4]; Sun et al., 2008[24]), electroluminescence (Vabbina et al., 2015[27]), fluorescence (Appel et al., 2005[2]), and colorimetric (Yamaguchi et al., 2009[31]; Sesay et al., 2013[21]) methods have been established for monitoring cortisol. From the above methods, only the colorimetric technique does not require an external instrument and the changing or appearing of color results can be observed by the naked eye. Enzyme-linked immunosorbent assay (ELISA) (Guo et al., 2009[12]; Upadhyay et al., 2018[26]) is based on an enzymatic reaction between enzymes on antibodies and a substrate providing high sensitivity of detection with enzymatic signal amplification. The cortisol ELISA kits are commercially available (for example, Cayman Chemical, USA and Diagnostics Biochem Canada Inc.). However, it requires several hours of analysis by a trained person and is limited by enzyme stability. Over the past decade, lateral flow immunoassay (Upadhyay et al., 2018[26]; Yamaguchi et al., 2013[30]) has been widely used for a screening test of antigens due to several advantages, such as ease of use and rapid detection. Almost all of the devices use a sandwich immunoassay. However, cortisol is a small molecule that would be difficult to implement by sandwich assay. Therefore, competitive immunoassay for cortisol has been developed with the different signal labeling materials of glucose oxidase (GOD) (Zangheri et al., 2015[33]) and horseradish peroxidase (HRP) (Chen et al., 2008[7]) conjugation of cortisol being reported. However, the substrate needs to use generating signals with multi-protocols of enzymatic reaction. In addition, commercial LFA with fluorescent (VerOFy®, Oasis Diagnostics® Corporation, USA) is available, however, it requires the portable fluorescence reporter (LIAM^TM^, Oasis Diagnostics® Corporation, USA).

Gold nanoparticles (AuNPs) (Ansari et al., 2010[1]; Jazayeri et al., 2016[14]; Banerjee and Jaiswal, 2018[5]; Cvak et al., 2012[9]; Choi et al., 2014[8]) have been widely employed as labels used for the immunoassay due to their strong optical absorption, good compatibility with antibodies, and long-term stability. Cortisol-BSA with AuNPs (Choi et al., 2014[8]; Leung et al., 2003[16]) and cortisol-3-carboxymethyloxime-adipic acid dihydrazide-bovine serum albumin (F-3-CMO-ADH-BSA) (Nara et al., 2010[19]) were applied for cortisol detection. However, using AuNPs has the limitation of sensitivity or requires complicated synthesis of cortisol compounds for competitive assay. More recently, the silver enhancer (Rodriguez et al., 2016[20]; Yeh et al., 2009[32]) for signal enhancement of AuNPs labels have been reported. By this method, AuNPs catalytic reduction of silver ions in the presence of a reducing agent resulting in participated silver ions on the gold nanoparticles surface is proposed. The enlarged particles become a dark color providing more intensity, so are more easily visualized.

Herein, we reported a competitive lateral flow immunoassay using AuNPs label with silver enhancement for simple and sensitive saliva cortisol testing. The color signal from AuNPs labeled antibody has an inverse relationship with the antigen concentration. By this approach, the sensitivity of the assay was improved by silver enhancement. The color results can be easily observed by naked eye and the cortisol levels were easily obtained by comparing with the color chart. The assay showed working ranges of 0.5 and 150 ng/mL and the sensitivity of 0.5 ng/mL. Therefore, this method is suitable for practical uses with rapid, ease of use, and portability for cortisol detection, which will be beneficial to indirectly prevent stress-related diseases.

## Materials and Methods

### Chemical and reagents

A mouse anti-cortisol monoclonal antibody, cortisol-BSA conjugate was purchased from Cusabio Biotech Co., Ltd, (China). Goat anti-mouse immunoglobulin G was obtained from Jackson ImmunoResearch Laboratories, Inc., Ethanol, disodium hydrogen orthophosphate, and sodium dihydrogen phosphate were purchased from BDH Prolabo (Lutterworth, UK). Hydrocortisone, 20 nm gold nanoparticles, citric acid, disodium citrate tribasic dehydrate, bovine serum albumin (BSA), and silver nitrate were purchased from Sigma-Aldrich (Missouri, USA). Hydroquinone was provided by Tokyo Chemical Industry Co., Ltd. Analytical-grade reagents and 18 MΩ-cm water were used throughout this experiment. The silver enhancer solutions were prepared by a 1:1 (V:V) mixing solution of 100 mg silver acetate in 50 mL milli Q water and 250 mg hydroquinone in a 50 mL of citrate buffer pH 3.8.

### Preparation and characterization of anti-cortisol antibody-conjugated AuNPs

Conjugation of anti-cortisol antibodies with AuNPs was performed by mixing of 100 µL of 175 µg/mL anti-cortisol antibody with 1 mL AuNPs pH 8, and followed by incubation under continuous stirring at room temperature for 30 minutes. After that, 100 µL of 3 % BSA in 20 mM PBS was added into a prepared solution to reduce nonspecific binding. The mixed solution was centrifuged at 12,000 rpm at 4 °C for 30 minutes. Finally, the separated precipitation was adjusted to the volume of 100 µL by 3 % BSA in 20 mM PBS buffer and stored at 4 ºC. Anti-cortisol antibody-conjugated AuNPs was examined and characterized under a Thermo Scientific NanoDrop 2000 Spectrophotometer and Transmission electron microscope (Hitachi/s-4800, Japan).

### Preparation of strip and procedure for competitive immunoassay

**Figure 1:**
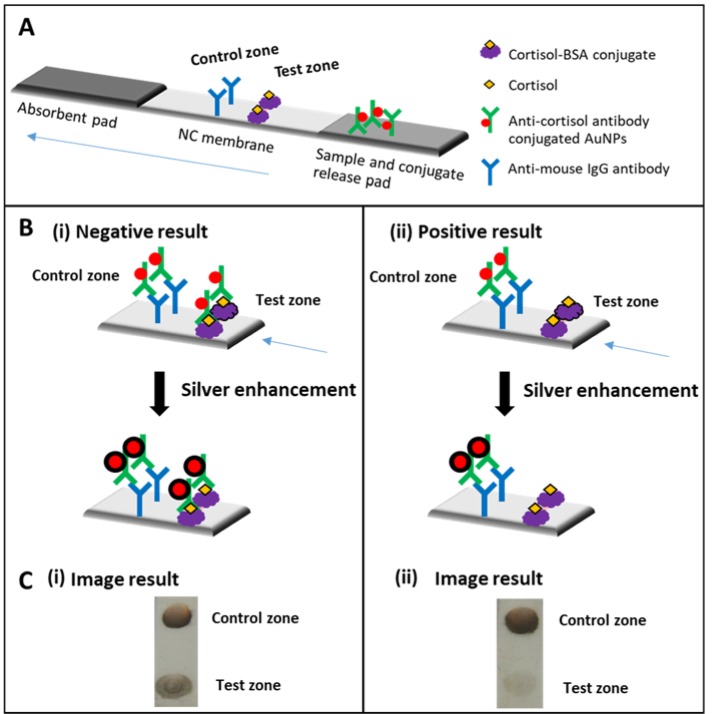
(A) Schematic drawings of lateral flow immunoassay based on competitive platform, (B) The principle of competitive assay at test and control zone on nitrocellulose membrane:(i) negative and (ii) positive results, and (C) image results of (i) negative and (ii) positive results

The schematic of the competitive lateral-flow immunoassay is shown in Figure 1A(Fig. 1). The test strip is composed of 3 components, which are a sample with a conjugate- released pad (Whatman FUSION 5™, GE Healthcare), a nitrocellulose pad (Whatman AE 98, GE Healthcare), and absorbent pad (Whatman CF6, GE Healthcare) stuck on a plastic backing card on which a sample load will pass. Anti-cortisol antibody conjugated AuNPs were immobilized on a conjugate released pad. The 1 µL of cortisol-BSA conjugate in 0.1 M PBS pH 7.4 and 1 µL of anti-mouse IgG antibody was dotted on the nitrocellulose pad as the test zone and the control zone, respectively and then dried for 1 hour at room temperature. 

For the detection step, the strip was dipped into 40 µL of a sample solution followed by 40 µL of 0.1 tween 20 in 0.1 M PBS pH 7.4 as the running buffer. After 2 minutes, the silver enhancer was added for 40 µL to complete assay. A negative result was indicated by a red and dark color at the test zone, before and after enhancement, respectively (Figure 1B, i(Fig. 1)). Whereas, a positive result presented pink to colorless according to their concentration (Figure 1B, ii(Fig. 1)). Both negative and positive results perform a dark color at the control zone. The test zones were taken using a digital camera (IXY200F, Canon, Japan) in automatic mode with no flash under a control light box. The image of (i) negative results and (ii) positive results were shown in Figure 1C(Fig. 1). After that, the images were exported to Adobe Photoshop CS6 program for semi-quantitative analysis. The color signal at the test zone was analyzed by measuring the mean intensity in the RGB channel. The mean intensity value of each test zone was obtained by subtracting the intensity from that of the background. Next, the background-subtracted intensity values were used to obtain a calibration curve.

### Analysis of salivary cortisol

Artificial saliva was purchased from Pickering laboratories (Mountain View, USA). Saliva samples were obtained from the volunteers in our laboratory. The study was approved by the Ethics Committee of Mahidol University, MU-CIRB 2017/ 231.1312. Saliva were collected at 8 A.M. before eating. Then the sample was centrifuged for 10 minutes at 4,000×g at 2-8 °C. The solution was then diluted 10 times in 0.1 Tween 20 + PBS pH 7.4 before testing. Salivary cortisol levels were measured using ELISA kit (Cusabio Biotech Co., Ltd, (China)) according to the manufacturer's protocol. 

## Results and Discussion

### Preparation and characterization of anti-cortisol antibody conjugated AuNPs

**Figure 2:**
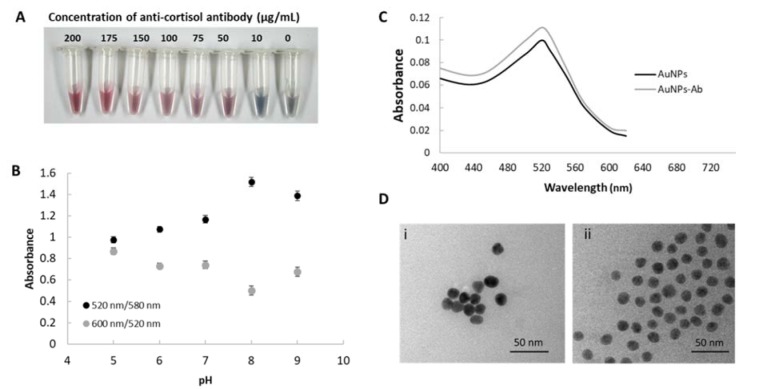
Optimization of anti-cortisol antibody and AuNPs conjugate: (A) image results of the AuNPs solution with a different concentration of anti-cortisol antibodies after adding of 10 NaCl, (B) Plot of absorbance at 520 nm versus pH of AuNPs after adding of 10 NaCl and a fixed concentration of anti-cortisol antibodies at 175 µg/mL, (C) UV-vis absorption spectra of AuNPs and anti-cortisol antibody-conjugated AuNPs (AuNPs-Ab) and, (D) TEM image of (i) AuNPs and (ii) anti-cortisol antibody-conjugated AuNPs.

Gold nanoparticles conjugated with antibodies influenced by the pH and antibody concentrations were optimized. Anti-cortisol antibody concentration was optimized by varying concentration at 10, 50, 75, 100, 150, 175 and 200 µg/mL. 20 µL of anti-cortisol antibodies were added into 200 µL of AuNPs then incubated for 15 minutes followed by adding 80 µL of 10 NaCl. The results are shown in Figure 2A(Fig. 2). In the presence of NaCl, the color of suspension of AuNPs changed into blue after addition of low concentration of anti-cortisol antibodies because of the aggregation of AuNPs. The anti-cortisol antibodies at above 175 µg/mL can prevent the aggregation of AuNPs because this concentration was enough for binding antibodies with AuNPs. Therefore, 175 µg/mL of anti-cortisol antibodies was used in further experiments. The effect of pH on anti-cortisol antibodies and AuNPs binding was studied from pH 5-9. The stability of colloidal solution and polydisperse was investigated using the calculated values from the ratio of absorbance at λ_max_/ 580 nm and 600 nm/ λ_max_ (λ_max_ = 520 nm) after adding of NaCl, respectively. The plot between absorbance ratio and pH was shown in Figure 2B(Fig. 2). The most stable and the least amount of polydispersion of AuNPs are at pH 8. Thus, the pH 8 was selected as the optimal condition for preparation of anti-cortisol antibody-conjugated AuNPs. The optical property of the unconjugated and anti-cortisol antibody conjugated AuNPs are shown in Figure 2C(Fig. 2). The UV-vis spectra of AuNPs and anti-cortisol antibody conjugated AuNPs exhibited maximum absorbance at a wavelength of 520 nm. After conjugation of anti-cortisol antibodies with AuNPs, the UV-vis absorption intensity slightly increased since the antibodies interacted with AuNPs. Additionally, the gold remained nanosize after conjugation from the Transmission electron microscope (TEM) image (Figure 2D(Fig. 2)). 

### Optimization of competitive assay

**Figure 3:**
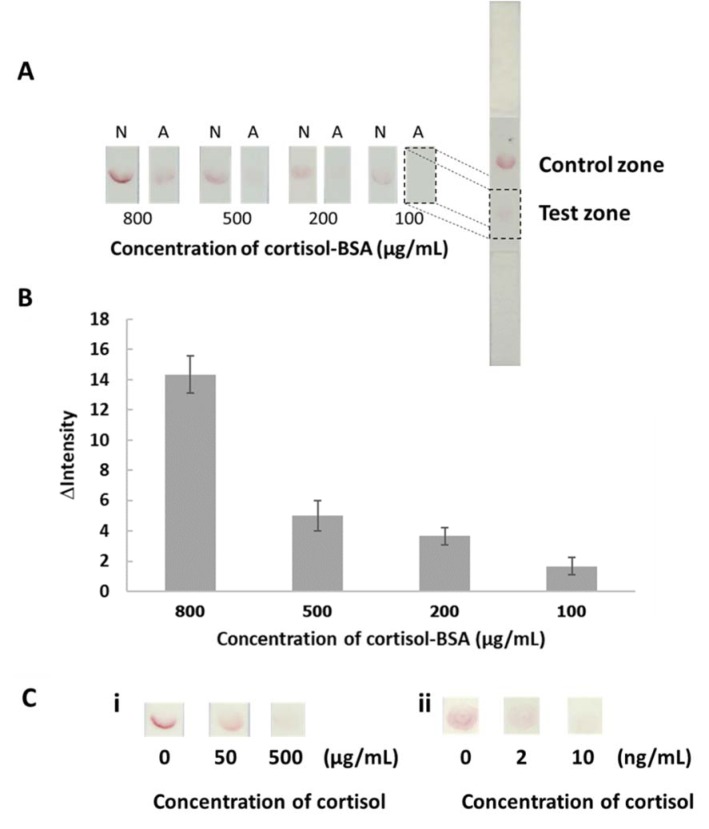
Effect of cortisol-BSA concentration on competitive immunoassay (A) Image of the results: N is negative control (without cortisol) and A is analysis, which contained 100 ng/mL cortisol, (B) plot of Δ intensity (intensity of the negative control - intensity of the analysis) versus cortisol-BSA concentration. The error bar is the standard deviation (n=3), and (C) Image results of competitive assay for cortisol detection using (i) 200 and (ii) 800 µg/mL of cortisol-BSA.

The detection of cortisol was based on a competitive immunoassay which is the cortisol in the sample competed with the fixed cortisol-BSA at the test zone. After application by analyst, the cortisol flow passes through the anti-cortisol antibody conjugated AuNPs to from a complex, which only the excess anti-cortisol antibody conjugated AuNPs are captured by immobilized cortisol-BSA on the NC surface at the test zone. The red intensity of the test zone is inversely related to the amount of cortisol, which allowed the cortisol to qualify. Parameters affecting the sensitivity of the competitive immunoassay on lateral flow immunoassay for cortisol detection were investigated. Firstly, the volume of pre-immobilized anti-cortisol antibody conjugated AuNPs on a conjugate release pad was varied and 3 µL provided the highest response of test (data not shown). Therefore, 3 µL of anti-cortisol antibody conjugated AuNPs was fixed onto the conjugate release pad. In addition, the pre-spotted cortisol-BSA concentration at the test zone was optimized at various concentrations from 0 to 800 µg/mL. In order to obtain the highest performance of the sensor for competitive detection, the different intensities of the signal obtained from negative control and positive results were investigated at the test zone. The strip was dipped into the analysis solution containing 0 and 100 µg/mL for negative control (N) and analysis (A), respectively. As shown in Figure 3A(Fig. 3), the visible red color on the test zone can be clearly observed with the cortisol-BSA concentration above 200 µg/mL. The highest difference in intensity was found at 800 µg/mL. Although, 800 µg/mL of cortisol-BSA provided a high intensity of negative control, the sensitivity of cortisol detection was lower than that of 200 µg/mL of cortisol-BSA, which was ng/mL levels as shown in Figure 3C(Fig. 3).

### Competitive assay with silver enhancement for cortisol detection

**Figure 4:**
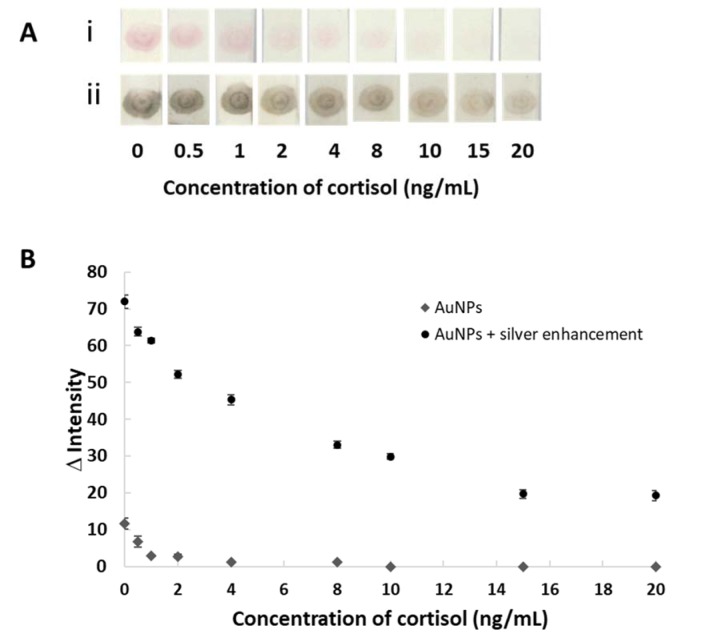
(A) The images on the test zone of various concentrations of cortisol at before (i) and after (ii) enhancement which were representative of those seen in three independent repeats of the experiment. (B) Plot of Δ intensity (intensity of the analysis - intensity of the background) determined by digital-image analysis using Adobe Photoshop (Red channel and RGB channel for AuNPs and AuNPs + silver enhancement, respectively). The data are derived from 3 replicates.

The cortisol detection was performed in the range of 0 to 20 ng/mL. The results of the competitive immunoassay for cortisol detection are shown in Figure 4(Fig. 4). Before enhancement, the red color was obtained at the test zone for the concentration of cortisol 0 ng/mL as the negative control (Figure 4A, i(Fig. 4)). The intensity was decreased with increasing the cortisol concentration and became colorless above 15 ng/mL. The limit of detection (LOD) by the naked eye was 2 ng/mL. However, the negative result (control) was difficult to distinguish by the naked eye. In order to enhance the signal, the silver enhancement solution was applied in the lateral flow immunoassay. After applying the enhancement solution, the red dots of the test changed to dark brown, which was related to the intensity of the red color (Figure 4A, ii(Fig. 4)). The reaction is based on the reduction of silver ions on the AuNPs's surface. The silver precipitate on gold provided the particle enlargement resulting in the color changing from red to dark brown. The results can be easily visualized within 5 minutes. The image picture of the results was taken at 5 minutes after applying silver. Figure 4B(Fig. 4) shows the plot of the mean intensity of the sensing area versus cortisol concentration with and without silver enhancement. Silver enhancement obviously improved the intensity of the results. The LOD by semi-quantitative analysis using an image processing program at 3 SD of blank + mean value was 1.8 and 0.5 ng/mL for without and with silver enhancement, respectively. Therefore, the sensitivity was enough to be applied for salivary cortisol detection.

### Analysis of salivary cortisol 

**Figure 5:**
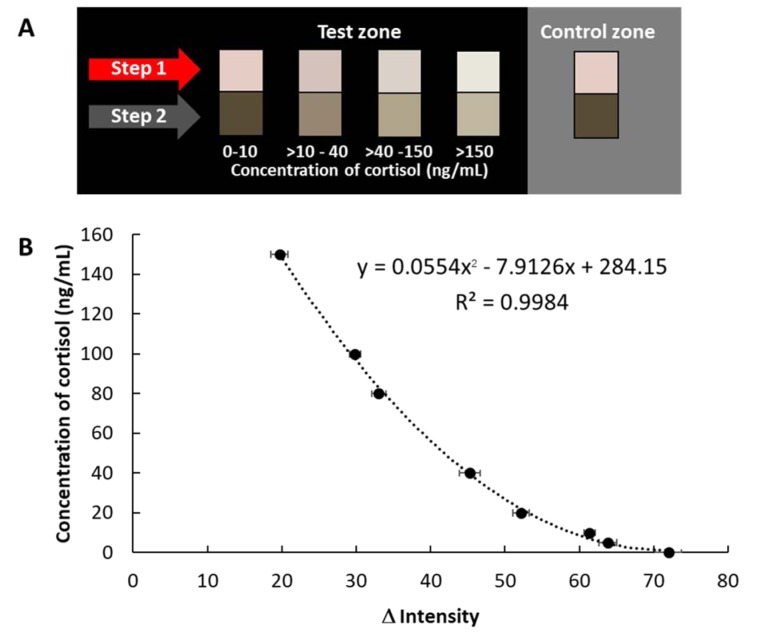
(A) The color chart of the cortisol levels, (B) Plot of concentration of cortisol (10-fold dilution) versus Δ intensity (intensity of the analysis - intensity of the background) determined by digital-image analysis using Adobe Photoshop. The data are derived from 3 replicates.

The assays are sensitive to viscosity, therefore, the dilution effect was investigated. The results obtained by 10 dilutions using 0.1 Tween 20 + PBS of saliva was comparable to the results obtained by cortisol in PBS. Additionally, the calibration curve (Figure 4 B(Fig. 4)) provided the working range from 0.5 to 20 ng/mL of cortisol test. Therefore, it can be applied for detection of salivary cortisol in diluted sample. A color chart has been constructed under optimized conditions with 10 times diluted saliva to qualify cortisol levels (Figure 5A(Fig. 5)). Moreover, the semi-quantitative analysis was performed using an image processing program in order to obtain more reliable measurements. The calibration plot (0.5-150 ng/mL cortisol) shown in Figure 5B(Fig. 5) uses the equation y = 0.0554x^2^ - 7.9126 x + 284.15, R^2^ = 0.9984. To investigate the performance of the developed assay in salivary cortisol determination, the approach was applied to the screening of cortisol in saliva and artificial saliva. The results are shown in Table 1(Tab. 1). The color results were compared to the color chart. The value of the cortisol was measured to be > 40 - 150, > 150, and > 150 ng/mL for samples 1, 2, and 3, respectively by visual measurement. For the semi-quantitative analysis by image processing, the cortisol value of samples 1, 2, and 3 were found at 112, 152 and 159 ng/mL, respectively (the cortisol value reported from previous studies were found to be about 29 -145 ng/mL (Suay et al., 1999[23]; Takai et al., 2004[25]; Westermann et al., 2004[28], for normal subjects). The cortisol levels depend on age, gender, health history and the method used for testing. In addition, the results obtained from the developed method were compared with the results obtained by ELISA kit (The equation was y = -5.0016x^3^ + 43.362x^2^ - 28.32x + 4.0087 and R² = 0.9999). A paired t-test at 95 confidence level was performed. The statistics revealed that t_calculated_ (2.33) was below t_critical_ (2.77) which suggests no significant difference between the two methods. This indicates a good agreement between the two methods.

Next, the recovery was studied by the spiked artificial salivary samples at 5, 15, and 50 ng/mL. The analytical recoveries ranged from between 68-106. The results indicated that this developed assay has sufficient performance for the rapid, sensitive and easy-to-use screening test of salivary cortisol. 

## Conclusions

The lateral flow immunoassay based on the competitive format coupled with silver enhancement system herein provides a simple and rapid screening method for cortisol detection. Measuring the samples requires only an addition of the sample and running buffer followed by the application of a silver enhancement (total assay was 15-20 min). The results can be observed by the naked eye or with an image processing program. The limit of detection was 0.5 ng/mL with the detection range at 0.5 to 150 ng/mL for salivary cortisol. The assay was successfully applied to the screening of salivary cortisol in a real sample. These findings demonstrated the potential application of a developed assay for clinical analysis for early prevention and monitoring of cortisol-related diseases.

## Acknowledgements

Authors wish to thank Mr. Bryan Kilvinski who assisted in the proof-reading of the manuscript. A.A. gratefully acknowledges support from Research Grant for New Scholar from the Thailand Research Fund (TRF) (Grant no. MRG6080078). A.A and O.C. greatly thank the Thailand Research Fund through Research Team Promotion Grant (RTA6080002).

## Figures and Tables

**Table 1:**
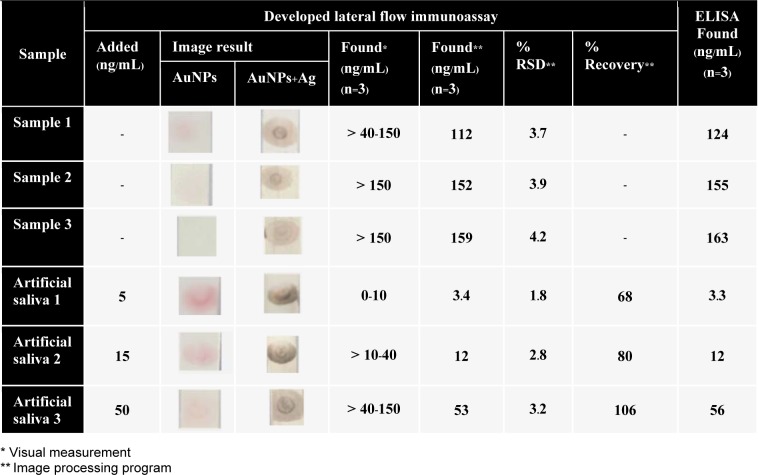
Determination of cortisol in salivary samples and spiked cortisol in artificial saliva at 5, 15, and 50 ng/mL compared with ELISA. The data are derived from 3 replicates.
